# Sexual and reproductive health of adolescent Syrian refugee girls in Lebanon: a qualitative study of healthcare provider and educator perspectives

**DOI:** 10.1186/s12978-021-01170-3

**Published:** 2021-06-06

**Authors:** Sasha Abdallah Fahme, Maia Sieverding, Sawsan Abdulrahim

**Affiliations:** Department of Health Promotion and Community Health, Faculty of Health Sciences, American University of Beirut, Beirut, Lebanon

**Keywords:** Refugee health, Adolescent health, Sexual and reproductive health, Early marriage, Sexual- and gender-based violence, Adolescent pregnancy, Global health

## Abstract

**Background:**

Adolescent Syrian refugee girls in Lebanon are thought to experience a disproportionate risk of poor sexual and reproductive health, related in part to conflict and displacement. The purpose of this qualitative study was to explore healthcare provider and educator perceptions of the sexual and reproductive health determinants and care-seeking behaviors of this vulnerable population. The findings of the study will inform a health intervention that aims to reduce early marriage and improve access to sexual and reproductive health information and services.

**Methods:**

In-depth interviews and focus group discussions were conducted with stakeholders who work with adolescent Syrian refugee girls in an under-resourced area of eastern Lebanon bordering Syria. Data analysis followed principles of Clarke and Braun’s thematic analysis.

**Results:**

Study participants perceived adolescent pregnancy, reproductive tract infections, and sexual- and gender-based violence as major population health needs. The study also identified a number of influencing structural and sociocultural determinants of health, including early marriage, adolescent disempowerment, and men’s disengagement from care. A conceptual framework based upon the Gelberg–Andersen Behavioral Model for Vulnerable Populations was developed to relate these determinants and guide pathways for potential interventions.

**Conclusions:**

Adolescent sexual and reproductive health interventions among Syrian refugees in Lebanon should adopt a multi-pronged, community-based approach to address underlying health determinants and engage with men and parents of adolescents. Special attention should be given to provider biases in healthcare settings accessible to adolescents, as these may reflect underlying tensions between host and refugee populations and discourage adolescents from seeking care.

## Plain language summary

Studies suggest that adolescent Syrian refugee girls in Lebanon are at risk of early marriage and related poor sexual and reproductive health outcomes, yet little is known about this population’s specific sexual and reproductive health needs and care-seeking practices. To help fill this gap, we conducted a qualitative study with healthcare providers and educators who provide adolescent Syrian refugee girls with sexual and reproductive health services and education. The study aimed to better understand this population’s health needs and behaviors to aid in the development of a sexual and reproductive health program for Syrian refugee girls that seeks to reduce early marriage. Respondents identified adolescent pregnancy, reproductive tract infections and sexual and gender-based violence as major health issues in this population. Early marriage, disruption of education, restrictive gender roles and men’s detachment from healthcare were all recognized as potential risk factors. Stigma and bias, even among the study participants themselves, were also found to be potential barriers to care. We adapted a behavioral model for healthcare-seeking practices among vulnerable populations to categorize these risks and develop recommendations for health interventions in this community. Sexual and reproductive health initiatives in this population should be multi-faceted, community-based and aimed not only at adolescents but also their spouses and parents, who hold decision-making power.

## Background

An estimated one million registered Syrian refugees reside in Lebanon, a country with the highest per capita refugee population in the world [1]. The majority of refugees, of whom 52% are female and 27% are girls under 18 [2], live in poverty [3]. As in many other contexts affected by conflict and forced displacement [4–6], Syrian refugee women and adolescent girls are highly vulnerable to negative sexual and reproductive health outcomes [7–17]. Qualitative studies have reported perceived increases in sexual assault, rape and intimate partner violence (IPV) within this population [12, 13, 15, 17]. Although rigorous studies of the prevalence of SGBV among Syrian refugees in Lebanon are lacking, a systematic review of exposure to sexual violence among refugees and internally displaced persons (IDPs) globally found that approximately one in five women in complex humanitarian emergencies had experienced sexual violence [18]. Again consistent with international evidence [19], some investigators have also drawn attention to the increased risk of engaging in “survival sex,” the practice of exchanging sex for essentials such as shelter and food, a known risk factor for sexually transmitted infections (STIs) [13, 15], among Syrian refugee women and children as young as ten years of age [12]. Studies of adolescent refugee girls in Lebanon [20], as well as multiple conflict-affected countries in Sub-Saharan Africa [4], have found that they have limited knowledge of SRH topics, including contraception. The risk of poor SRH may be further exacerbated by limited access to healthcare [15, 17]. Among Syrian refugees, high costs, insufficient services, fear of discrimination and poor reproductive health literacy have all been identified as factors hindering access to SRH care [12, 15, 17, 21].

Early marriage, defined as marriage prior to the age of 18, may be a significant driver of poor SRH among adolescent Syrian refugees in Lebanon. Early marriage disproportionately affects girls in disadvantaged communities who are more likely to drop out of school, and is associated with negative health consequences, including those related to adolescent pregnancy [11, 22]. The global evidence indicates that whether conflict results in increased or decreased rates of early marriage is context-specific [6]. Among Syrian refugees in Lebanon, a number of reports and qualitative studies have suggested a rise in early marriage since displacement [15, 17, 23, 24]. For example, Bartels and colleagues estimated that 35% of Syrian girls under 18 years of age in Lebanon are married, a figure roughly four times higher than that in Syria prior to the conflict [23]. Notably, there were subnational differences in early marriage rates as well as SRH outcomes in Syria prior to the conflict [25, 26]. Syrian refugees who were displaced to neighboring countries are also a selected population that is not representative of the national pre-war Syrian population. Of particular importance, Syrian refugees in Lebanon have lower rates of educational attainment than the national pre-war population, as well as than the Syrian refugee population in other neighboring countries such as Jordan [27]. The refugee population in Lebanon is also younger, with a higher percentage of children, and more female than the populations of refugees who went to further destinations in Europe [28]. As a result of these dynamics, previous studies in Jordan have demonstrated that the high rates of early marriage among Syrian refugees are driven by selective migration from certain regions and disadvantaged socioeconomic strata in Syria where rates of early marriage were high prior to the conflict, rather than increases in early marriage post-displacement [29]. Comparable studies are not available in Lebanon. Nevertheless, the high prevalence of early marriage among Syrian refugees in Lebanon suggests that there is a large population of adolescent girls in need of SRH services. The young age structure and disadvantaged educational background of refugees further points to the importance of understanding SRH outcomes among this population.

The combination of early marriage and disruptions in access to contraception in emergency contexts, including those of forced displacement, may also influence women’s risk of unintended pregnancy [30, 31]. This may then carry far-reaching implications on both maternal and neonatal health, particularly in a patriarchal and conservative society such as Lebanon, where abortion is criminalized under most circumstances [32]. Studies of the impact of conflict on fertility in culturally comparable settings such as Iraq [33] and the West Bank and Gaza [6, 34, 35] have shown increases in adolescent fertility of up to 700% during times of armed conflict, thought to be driven primarily by early marriage. However, an analysis of Palestinian refugees in Jordan did not observe such marked increases in fertility [6, 35], highlighting potential differences in contraceptive utilization between conflict-affected populations who remain in their country of origin and refugee populations. Importantly, access to SRH services for refugees is dependent on host country factors, including access to and the organization of host country health systems and residence in camp versus community settings, as well as on experiences of conflict [36, 37]. For instance, only 35% of Syrian women in Lebanon reported using contraception, as compared to approximately 60% of women in pre-conflict Syria [14, 17]. The lower rate of contraceptive use among Syrian women refugees may be due to a combination of factors including the selectiveness of the refugee population, cost and other barriers to accessing contraceptives in Lebanon, and changes in fertility intentions in displacement.

### Healthcare providers and educators as key informants for adolescent SRH

Healthcare providers, teachers, and school social workers are key informants who come into contact with adolescents and their parents, and who can provide an important perspective on the SRH information and services available to Syrian adolescents in displacement. Information on puberty and SRH is sometimes incorporated into school curricula and health services may be provided at or in coordination with schools [38]. Teachers and school social workers may learn about girls’ SRH needs during sessions on puberty and menstruation, which in Lebanon are typically presented in eighth grade biology class, or through regular interactions with adolescents in the school setting. Thus, they are a potentially important source of referrals to care as well as information and advice for adolescents who remain in school.

When adolescents seek SRH services, they are likely to come into contact with frontline healthcare providers, including nurses, midwives and doctors. Like educators, healthcare providers have a first-hand perspective on the SRH needs of this population, including those adolescents who are not in school but present to medical care. Moreover, providers’ attitudes towards adolescent SRH care-seekers and the completeness and accuracy of information that they give adolescents are critical for SRH outcomes [39]. If adolescents have negative experiences seeking SRH services or are unable to obtain the services they desire, it may discourage them from future care seeking. Provider perspectives on adolescent SRH needs are thus important both because of their experience with this population’s needs and because their perspectives may shed light on some of the challenges that refugee adolescents in the Lebanese context face when trying to access SRH services.

The purpose of the present qualitative study was to explore the experiences and perspectives of educators and healthcare workers who provide SRH information and/or services to adolescent Syrian refugee girls in Lebanon. Specifically, the study aimed to: (i) explore these stakeholders’ beliefs regarding the determinants of SRH in this population, types of SRH information and services accessible to Syrian adolescents, settings in which such information and services are typically disseminated, and challenges encountered when providing adolescent SRH information and care, and; (ii) identify ways in which a planned early marriage intervention could engage with the Syrian refugee community to address adolescent SRH needs. We applied the Gelberg–Andersen Behavioral Model for Vulnerable Populations, a framework which incorporates intersectional vulnerabilities in examining disparities in healthcare accessibility and care-seeking behaviors among marginalized populations, to characterize and relate our findings within the broader context of Syrian displacement in Lebanon [40].

This study is part of a larger implementation research study, Project Amenah, which aims to mitigate the drivers of early marriage among adolescent Syrian refugee girls living in Lebanon through enhancing their access to SRH information and services. Building on a 2016 prevalence survey of early marriage in these communities [41], Project Amenah began in 2018 as a community-based, multi-component, pilot intervention that focused on 11–14-year-old Syrian refugee girls enrolled in school within the Bekaa governorate of Lebanon. Adopting an ecological approach that conceptualizes the home and school as two settings strongly influencing adolescent development, the pilot intervention consisted of life skills sessions with girls and discussion sessions with mothers aimed to empower girls and promote retention in education. The sessions were delivered by community workers from the refugee community over a period of five months. Our experience in this pilot study showed that among adolescent refugee girls, there was a knowledge gap around fundamental aspects of SRH, including puberty and menstruation. The next phase of Project Amenah seeks to address this gap by incorporating a more explicit focus on SRH. In conjunction with additional formative work, including a literature review of SRH interventions in the Arab world and a qualitative study with adolescent Syrian refugee girls themselves, the present study with stakeholders was intended to help inform the development of this upcoming intervention.

## Methods

### Study setting

The data for this study were collected in the Bekaa Governorate in eastern Lebanon. This region was chosen as it has the highest population of Syrian refugees in Lebanon and was the setting of the Amenah Project and the 2016 early marriage prevalence study mentioned above, which demonstrated that 24% of Syrian refugee girls below age 18 were married [41]. Due to its proximity to the Syrian border, the Bekaa Governorate has a high density of refugees and poor socioeconomic conditions that may help foster the practice of early marriage [42].

### Participants and data collection

A purposive sampling strategy was adopted to identify stakeholders working in the health and education sectors who interacted with adolescent Syrian refugee girls. We generated a list of local health organizations that provide services to Syrian refugees and schools where Syrian adolescents receive formal education. Two clinics located in the study area were identified as recruitment sites based on the fact that they serve a majority Syrian refugee patient population and provide subsidized SRH services. Three schools were similarly selected based on their location and student population of adolescent Syrian girls. Eligibility criteria for individual participants included age above 18 years, primary employment in the identified institutions, and professional interaction with adolescent Syrian refugee girls. Following formal administrative approvals from all the sites, we invited teachers, principals, school social workers, and healthcare providers who met the selection criteria to participate in the study.

In April and May of 2019, the study team conducted four individual in-depth interviews (IDIs) (three with educators and one with a healthcare provider) and three group interviews with two participants each (one with educators and two with healthcare providers). Moreover, the study team conducted two focus group discussions (FGDs), one with educators (3 participants) and one with healthcare providers (5 participants). Group interviews and FGDs were organized by profession in order to facilitate discussions specific to participants’ domain of expertise. The decision of whether to conduct an in-depth interview or FGD was based upon participant availability. In total, the study included 18 study participants of both Lebanese and Syrian nationalities and of whom the majority (N = 17) were women. Study participants represented a fairly homogenous population, which facilitated achieving data saturation. Eight of the participants were educators, including teachers, school social workers and principals. The nine healthcare professionals who participated included midwives, nurses and obstetrician-gynecologists.

Interviews and FGDs were conducted in Arabic and led by the paper’s first author, a physician with experience providing clinical care to refugees. Semi-structured interview guides were developed based on unpublished formative research on early marriage and SRH outcomes of adolescent Syrian refugee girls conducted by the study team, as well as a literature review of SRH educational interventions in the Middle East and North Africa. Topics explored in the interview guides included perceptions of the adolescent Syrian refugee population’s SRH needs, determinants of adolescent SRH outcomes, and where and how adolescent refugees access health information and services. During the interview, stakeholders were also asked to offer recommendations on how to develop an effective SRH intervention in this community. Ethical approval for the study was obtained from the Institutional Review Board of the American University of Beirut in Beirut, Lebanon.

### Theoretical framework and data analysis

In-depth interviews and FGDs were recorded using a digital audio recorder and transcribed verbatim into Arabic. Data analysis followed principles of Clarke and Braun’s thematic analysis, which specify seven distinct stages of data analysis, including: familiarization with data (which occurs prior to and following transcription), transcription, creating initial codes, developing themes, reviewing themes, defining and naming themes, and writing a report [43]. The first author became familiarized with the data by listening to the audio recordings following each interview or FGD, reading transcripts, and developing a preliminary list of codes using Dedoose software V 8.12.14 (Los Angeles, CA: SocioCultural Research Consultants, LLC). Applying an inductive approach, codes were derived from the content of the transcripts and were simultaneously generated and grouped. All elements of the data were regarded with equal importance during this process. For example, one code family that we initially developed was “barriers to contraception use”, which captured descriptions of primarily sociocultural challenges to family planning and modern contraceptive accessibility among adolescent girls. We had originally grouped several sub-codes under this family, including “masculine identity as a barrier to care”, “husbands do not accompany wives to clinics”, “male authority over women”, “contraceptives restricted to married adolescents”, “conservative society”, “involvement of female relatives in adolescent SRH care”, and “premarital sex unaccepted”. Yet it soon became clear from the data that there existed distinct challenges in contraception accessibility for married and unmarried female adolescents. The former faced greater barriers from within their households (i.e. from their spouses and/or female relatives), whereas both married and unmarried adolescents faced environmental barriers related to gender roles. To reflect these differences, the code family was separated into two separate code families: “household barriers to contraception accessibility”, which captured data primarily about married adolescent girls, and “societal barriers to SRH care accessibility”, which reflected data pertaining to both married and unmarried adolescents. During still later stages of analysis, both groups were aligned under the heading of “disempowerment of adolescent girls”, which we identified as a major sub-theme of this research. In a similar fashion, all codes were extensively reviewed, ultimately generating a list of themes, which were reviewed and named. The themes developed were comprehensive, and checked against each other and the original data. Illustrative quotations were selected and translated into English. All quotations were reviewed against the original transcripts for accuracy.

The Gelberg–Andersen Behavioral Model for Vulnerable Populations [40] was used as a theoretical framework to interpret the identified themes. This model has been previously adopted to evaluate health practices, access, and disparities among disadvantaged communities including U.S. domestic homeless and incarcerated populations [40, 44, 45]. For a comprehensive description of the model and an example of its application to marginalized populations, we recommend Gelberg, Andersen, and Leake [40]. The framework builds upon the original Behavioral Model developed in the 1960s by expanding on “Traditional” individual-level factors to include predictors related to vulnerability [40, 46]. These are further organized into: (i) “Predisposing Vulnerable” factors, which include characteristics that may occur prior to disease onset and that are related to social structure such as immigration status, gender, living conditions, and victimization; (ii) “Enabling Vulnerable” factors, which are those individual- and community-based factors that may facilitate or impede health services utilization and include competing needs, social support, coping mechanisms, and availability of services, and; (iii) a “Need Vulnerable” domain which includes perceived and confirmed health needs related to vulnerability such as STIs, tuberculosis, and substance use disorders [40, 44–46]. These three major headings were applied to our data and subsequently adapted into a conceptual framework that further distinguishes between structural and sociocultural predisposing health determinants, delineates relationships, and guides pathways for potential interventions.

## Results

The main themes and sub-themes that were developed from the data are presented in Table 1. The three themes align with each of the main population characteristic domains described by the Gelberg–Andersen Behavioral Model for Vulnerable Populations. We describe each theme and its subthemes narratively, followed by a presentation of a conceptual framework and potential points of intervention.

**Table 1:** The major themes and sub-themes identified in the study

Themes	Sub-themes
Perceived adolescent SRH needs	Adolescent pregnancy
	Reproductive tract infections
	Sexual and gender-based violence
Predisposing determinants of adolescent SRH	*Structural determinants*
	Disruption of education
	Early marriage
	Limited healthcare resources
	*Sociocultural determinants*
	Adolescent disempowerment
	Stigmatization of premarital sex
	Limited SRH-related communication
Enabling drivers of adolescent SRH	Poor SRH knowledge
	Men’s disengagement from care

### Theme 1: perceived adolescent health needs

Healthcare providers agreed that adolescent Syrian refugee girls carry a high burden of poor SRH. According to study participants, the major population health needs included adolescent pregnancy, reproductive tract infections (RTIs), and complications of SGBV.

#### Adolescent pregnancy

Among Syrian refugees, adolescent pregnancy takes place primarily within the context of early marriage. Study participants indicated a clear need for contraception and family planning among adolescent refugees, and reported that clinics in the area provide a wide range of free or low-cost contraceptives, including oral contraceptive pills, intrauterine devices, injectable contraceptives, implantable contraceptives, and male condoms. Emergency contraception is also available, though its use is typically limited to cases of rape.

Yet not all sexually active adolescents who wish to prevent pregnancy may have access to these facilities and methods. There are a number of barriers rooted primarily in sociocultural gender norms, discussed in greater detail in the following section, which preclude adolescents from accessing contraception, particularly if they are unmarried. As a result, participants said that some women resort to abortion to terminate an unintended pregnancy. As abortion is illegal in Lebanon except under very limited circumstances, healthcare providers stated that women may resort to ineffective or unsafe abortive remedies at home that may put their health and life at risk.

“I know of some [refugees] who have home remedies for abortions that they use to terminate unintended pregnancies.” (Midwife, FGD).

It is not clear from our data the extent to which termination of pregnancy is common amongst Syrian adolescents or associated with poor access to contraception.

#### Reproductive tract infections

Healthcare workers participating in this study believed that there was a high burden of RTIs among adolescent refugee girls although their beliefs are not based on evidence given that limited diagnostic capacity at most healthcare facilities precluded the determination of the exact prevalence of specific infections, including those RTIs that are sexually transmitted. Although participants noted that poor awareness of RTIs among providers may make it difficult to appropriately identify and treat all cases, several postulated that vaginal trichomoniasis was likely the most common STI in this population. Healthcare providers who believed that Syrian women are at high risk of STIs stated that they screen all women irrespective of the reason for the medical visit.

“Patients won’t specifically present for STIs. So, I’ve trained [my staff] to screen all patients for STI symptoms. This is because some of the midwives may not realize that [Syrian refugee] women are vulnerable to STIs.” (Midwife, IDI).

The prevalence of STIs among Syrian refugee women and adolescents in Lebanon has not been studied and remains unknown. Bio-behavioral surveillance studies examining the prevalence of STIs and potential high-risk behaviors are needed to confirm the perceptions elicited in this study and improve our understanding of this population’s sexual health risk.

#### Sexual and gender-based violence

Stakeholders working in both the education and health sectors emphasized the pervasiveness of sexual violence among adolescent refugee girls. Primary and middle school social workers described several instances in which Syrian girls privately shared with them personal experiences of violation. Such disclosures typically followed classroom lessons and discussions about human rights.

“A scenario I might teach is… ‘A strange man tries to put his hand in your hair, for instance, but it’s your hair, and it’s a part of your body. And no one is allowed to touch any part of your body, because this is how people begin to take advantage of you. They may use this as an opportunity to cause you harm’…There are many [refugee] girls who are particularly mindful of these lessons. I feel like they pay special attention when I give these lessons, as though they are thinking ‘Yes’, like they have personally experienced these scenarios. For instance, a girl will come up to me privately after the lesson and say ‘Miss, what you were saying is very true, because something similar happened to me’…This means that they are being exposed to harm and exploitation.” (School social worker, FGD).

Some clinics available to refugees in the study area have established protocols for SGBV cases that include post-exposure prophylaxis, hepatitis B vaccination, pregnancy testing, emergency contraception, empiric STI treatment, and referral to legal and psychosocial services. Yet despite the availability of such resources, study participants said that there are likely to be even more adolescents and women exposed to SGBV who do not present for care, due to fear, shame, and limited awareness of psychosocial and health services.

“Truthfully, there aren’t many cases [of rape] in our clinic, but I think there are many [unreported] cases in the community. They don’t come here…I’m not sure if [women in the community] don’t know about the [sexual assault] services we provide.” (Midwife, IDI).

Though a number of qualitative studies have described an increase in IPV among Syrian refugee women related to severe poverty, the disruption of traditional gender roles, and systemic discrimination by host communities [12, 15, 17, 23], no studies to date have quantified this risk or explicitly focused on adolescent Syrian refugee girls as a distinct group with unique vulnerability to SGBV.

### Theme 2: predisposing determinants of health

Participants brought into focus a number of predisposing vulnerable domains that were perceived to influence the SRH of Syrian refugee women and adolescents in Lebanon. We further classified these domains into structural determinants, which are rooted in socioeconomic and political inequities, and sociocultural drivers, which reflect gender dynamics and societal practices and beliefs. We present each cluster of determinants separately in this section while acknowledging that structural and cultural determinants are often difficult to disentangle from one another.

#### Structural determinants

##### Effects of conflict and displacement on education

Conflict and displacement may have indirectly had a negative impact on the SRH of the Syrian refugee population by curtailing opportunities for education. Study participants cited dire socioeconomic circumstances, overcrowded living conditions, and limited resources as the primary reasons why refugee children might be withdrawn from school, usually to marry or find employment. They stated that exclusion from schooling affected adolescent girls’ health in several ways. First, out-of-school adolescents are inherently excluded from classroom-based SRH curricula, which are typically administered between the 7th and 12th grades in Lebanese schools. Second, even Syrian adolescents attending school may not be exposed to SRH curricula, as the school day for many refugees is shortened and condensed into an afternoon session, during which time other subjects are prioritized. Adolescent refugees therefore often rely upon their family, specifically mothers or female relatives, for information on SRH.

“Schools are the most important place where students can learn about SRH … We start [a curriculum on menstruation, personal hygiene, and puberty] at age 11–12, around the time of puberty for most girls…but for those girls who are not in school, it becomes their parents’ responsibility to teach them.” (School social worker, Group interview).

However, mothers of adolescent girls are often an unreliable or incomplete source of information, as they themselves have limited SRH knowledge and may unknowingly pass along inaccurate information to their daughters.

“The issue too is that the mothers are not educated on [SRH] and so they are unable to provide accurate and meaningful information to their daughters.” (Primary-school teacher, FGD).

Thus, given their parents’ backgrounds and their own limited access to schooling in Lebanon, most adolescent refugee girls are left with a poor basis of SRH knowledge, which may carry significant implications for their health.

##### Early marriage

In the cultural context of Syrian refugees in Lebanon, early marriage was identified by study participants as a major determinant of poor SRH among Syrian refugees in Lebanon. Early marriage in this population is not strictly the product of displacement, but rather can be considered as the outcome of intersecting structural and sociocultural factors. Additionally, it is often a necessary prerequisite to adolescent pregnancy in this context, and reflects a growing social pressure on women in this community to bear multiple children as a means of compensating for the untimely death of loved ones during the war.

“There are a lot of influencing factors [related to adolescent pregnancy] …A lot of people lost their sons and daughters in the war and are trying to have more children to make up for these deaths.” (Midwife, IDI).

Respondents identified multiple factors thought to be contributing to early marriage in this population. While some study participants attributed early marriage to cultural norms, the more salient view identified this practice as a mechanism to overcome some of the sociopolitical and economic consequences of conflict and displacement. These included poverty, perceived lack of security, overcrowded living conditions, limited educational opportunities, and a shifting collective consciousness toward marriage, which increasingly is regarded as an obligation, rather than a celebration.

“Poverty is the number one cause of early marriage here…In Syria, there were cases [of early marriage] for sure, but usually something you only saw in isolated, remote communities. Here, it has become the standard. It is a woman’s duty to marry when she becomes 14 years of age. This is because of poverty; families say, ‘Well, I can’t support her any longer’, and so their daughters get married.” (Obstetrician-gynecologist, Group interview).

Though there is little to no evidence to corroborate that early marriage was rare and limited to certain communities in Syria before the conflict and that it has become normative following displacement, such beliefs are nonetheless pervasive. While the methodological challenges of comparing early marriage and its determinants in pre-conflict Syria to post-displacement limit our ability to make inferences on the extent to which this practice has increased in Lebanon, poverty related to displacement was suggested by participants as a potentially important structural driver of early marriage in this context.

##### Limited healthcare resources

There is a paucity of adolescent-friendly, high-quality, low-cost primary SRH care available to refugees. Though marriage theoretically grants adolescent girls’ access to SRH services, a number of barriers practically limit their accessibility. Local and international humanitarian NGOs are the main providers of SRH care and education in the study area. These organizations may have unpredictable sources of funding, which in turn impacts their delivery of goods and services. For instance, one of the major centers for refugee women’s SRH care in the Bekaa, a Syrian-led NGO that depends largely upon foreign donations, was no longer able to offer free long-term contraception and was altogether unable to provide barrier contraception due to budgetary constraints at the time of the data collection. Participants also critiqued NGOs or poorly trained providers for distributing contraception without appropriate counseling.

“Once I remember an NGO had distributed condoms in one of the camps…but didn’t teach [the residents] how to use them… [The residents] thought they were balloons. Some people washed them.” (Midwife, FGD).

Healthcare providers highlighted limited capacity for diagnostic testing of RTIs, another important structural barrier to SRH care. An alternative “syndromic” approach is instead adopted in which women are empirically treated for multiple possible RTIs, including STIs. Patients are sometimes provided with empiric pharmacotherapy for their partners, but this practice is provider- and clinic-dependent. Furthermore, there is no infrastructure for surveillance or follow-up of either the patient or her partner to ensure eradication.

“Sexually transmitted infections are common but we have no way of knowing for sure. Because we don’t test for them, and because symptoms of STIs and RTIs are similar, we have no way of knowing if a woman has an STI or an RTI…So we empirically treat any woman who presents with symptoms. We also provide them with medications for their husbands. But we don’t know if [the husbands] actually take the treatment.” (Obstetrician-gynecologist, Group interview).

Empiric treatment of STIs has cost implications in a low resource setting and may affect emerging antibiotic resistance in this population. Furthermore, the lack of diagnostic capacity may carry implications for non-curable STIs, including HIV and human papilloma virus, the prevalence of which remain unknown in the Syrian population.

#### Sociocultural determinants

##### Limited SRH-related communication

Discussing matters related to SRH with adolescent girls is considered taboo in Arab culture. Parents generally believe that it is better for girls not to learn about their sexual and reproductive functions until they are about to marry. Nearly all study participants agreed that mother-daughter communication on matters related to SRH is strained and cited personal discomfort, shame, and stigmatization of adolescent sexual activity as possible barriers to dialogue.

“Parents don’t bring up these topics [with their children]. They’ll tell you: ‘You’re opening up my daughter’s eyes to something bad!’ Or they’ll say: ‘She won’t be thinking about these things until she hears about them.’ But who says she’s not thinking about this? Maybe she is thinking about these topics but she is too afraid [to discuss with her parents]? If a mother is unable to educate her daughter [on this issue] then she should ensure that her daughter is receiving the correct information elsewhere.” (High-school teacher, IDI).

Study participants additionally suggested that distrust between mothers and daughters could impact their willingness to discuss sensitive issues.

“I get the sense that [adolescent refugees and their mothers] do not have these types of relationships where they trust one another enough to discuss these topics.” (Primary-school teacher, FGD).

It is unclear whether mother–daughter communication on SRH-related matters has declined due to displacement or whether this is strictly a cultural norm, as there are no available studies or reports on this topic in pre-conflict Syria.

##### Disempowerment of adolescent girls

Women’s reproductive empowerment, conceptualized as “both a transformative process and an outcome”, encompasses women’s active and independent participation in both their sexuality as well as informed SRH decision-making [47]. Adopting this definition, study participants perceived adolescent Syrian girls to be disempowered, as they are generally restricted from governing their own SRH needs.

Participants suggested that men in this community typically oversee their adult wives’ use of contraception, and that such a dynamic is further complicated when they are married to an adolescent girl, whose healthcare accessibility is additionally determined by older female relatives, including mothers-in-law. This authority, reinforced by patriarchal gender roles, prioritizes the potentially conflicting interests of other parties over those of the adolescent herself.

“I had [an adolescent] patient come in with her mother, who told her that if she were to use contraception and delay her first pregnancy, her husband would leave her. When I offered her contraception [anyway], her mother became very angry and asked me how I could do such a thing. I told her that [the patient] asked me for it and that her husband was amenable. But her response was that [the patient’s] husband would marry someone else.” (Midwife, FGD).

Nearly all study participants agreed that men oftentimes impede their adolescent wives from accessing SRH care by prohibiting contraceptive use. In these cases, adolescents may ask their peers to obtain contraception on their behalf, as they themselves are unable or unwilling to present to a clinic.

“A lot of people come into our clinic asking for contraception for other women in their communities because their husbands don’t allow them to use contraception.” (Midwife, FGD).

Though clinics do not require third party consent to provide contraception to adolescents or adult women, stakeholders agreed that married women, irrespective of age, in practice need their husbands’ approval prior to initiating contraception.

“The other day, a woman came in asking for Implanon even though her husband refused to let her use contraception. I placed it. About two months later, her husband was hitting her and felt the device in her arm. He told her to come in to get it removed.” (Midwife, FGD).

Stakeholders identified several factors that may influence men’s decision-making around contraception. Firstly, gender norms are instilled in boys at an early age that establish them as decision-makers over female members of the household. Secondly, participants described a link between masculinity and fertility in the community they serve, expressing that many men associate masculinity with the ability to father children, and that such notions of male identity are established at a young age.

“A man [in this community] defines his manhood by this exact issue. He is only a man if his wife is able to become pregnant. And if she is unable to become pregnant, it’s as though he is not a man.” (Health teacher, FGD).

It is possible, therefore, that adolescent girls who are unable to advocate for their needs and negotiate contraceptive use are inadvertently coerced by their spouses and other relatives into becoming pregnant. Though most adolescent girls are thought to marry men who are also very young, participants perceived that the opinions and medical decision-making capacity of adolescent girls—but not their young spouses—in this community are inherently undervalued, such that girls are disproportionately disempowered and prevented from controlling their own health needs.

##### Stigmatization of pre-marital sex

Societal attitudes toward gender, sex, and marriage may restrict adolescent access to SRH education and care in several ways. Stigmatization of pre-marital sex, particularly for girls, may discourage unmarried adolescent girls from seeking healthcare altogether, even when SRH services are available to them.

“Of course [pre-marital sex] happens…But we don’t see it in our clinic. [These adolescents] care mostly about confidentiality and in our clinic, they might recognize someone they know, which…keeps them from coming here.” (Obstetrician-gynecologist, Group interview).

The physician further described how mothers went out of their way in order to hide that their daughters needed to be checked for an SRH concern:

“[Unmarried adolescent girls] come in with their mothers, who will list their [the mothers’] names at the front desk, or someone else’s name altogether. Only after they come in to see me will they admit that they are there for their daughter. They’re embarrassed. It’s not accepted in our society.” (Obstetrician-gynecologist, Group interview).

Unmarried adolescents’ apprehension to discuss their sexual activity may present a challenge to providing appropriate care and also to estimating the burden of STIs in this population. As many STI symptoms mimic those of non-sexually transmitted RTIs, a thorough sexual history is critical for determining the accurate diagnosis and therapy, particularly in resource-constrained settings that lack formal diagnostic capability.

“In the setting that an unmarried girl presents with symptoms concerning for a sexually transmitted infection, she never openly admits to engaging in sexual activity.” (Obstetrician-gynecologist, Group interview).

Although healthcare workers and educators critiqued parents and cultural norms in the community, they themselves also expressed personal and professional biases against providing SRH information and services to unmarried adolescents. One of the physicians interviewed reported offering IUDs only to adolescents who were both married and had had at least one pregnancy. Restriction of contraception in this way, whether implicit or overt, appears to be a normative practice among some of the healthcare providers interviewed in this study, and relates to both deeply prescribed gender roles and societal pressures regarding pregnancy, as well as providers’ non-evidence-based medical opinions. Similar biases were observed among educators as well.

“[Unmarried and married refugee girls] should all receive the same information. The only difference is that you should be clear about when it is permissible to have sex. The married adolescents can, the unmarried can’t. None of the major religions allow pre-marital sex, and nor do our own morals.” (School principal, Group interview).

Such beliefs, which were common among study participants, continue to reinforce the stigma surrounding pre-marital sex, which disproportionately restricts girls’ right to good SRH. The prevalence of such views within the community and among key providers of SRH services and information may deter unmarried adolescents from seeking care.

### Theme 3: enabling drivers of adolescent sexual and reproductive health

Study participants identified a number of enabling vulnerable domains that may impact adolescent SRH in this community. These are potential drivers that positively or negatively influence health outcomes by more directly impacting accessibility and/or utilization of care. Further, these drivers are related to, and in some cases may emanate from, the previously described predisposing determinants.

#### Poor SRH knowledge and misconceptions

Study participants expressed their belief that low SRH knowledge disproportionately affects adolescent girls, who are perceived as being likely to depend upon unreliable sources of SRH information. Stakeholders described adolescents learning about contraception from peers through misinformed exchanges that generate confusion and foster doubt toward family planning. Such misconceptions may have potentially devastating effects on adolescent girls’ and women’s health, as misinformation may directly affect care-seeking behavior.

“It depends on what [the patients] have heard [about contraception] before they’ve come in. For instance, if her neighbor has an IUD and she’s talked to her about it, she’ll come in requesting an IUD.” (Midwife, FGD).

“We have had women coming [to the clinic] several days after getting Implanon requesting that it be removed because their husbands have heard that it causes cancer, or that it can migrate under the skin and embolize to the heart. Even if their husbands were initially accepting of the contraception, they may have heard from their friends or others that it poses health risks to the woman.” (Midwife, FGD).

Among young married adolescents, a fundamental lack of SRH knowledge may exacerbate the balance of power dynamics within the context of their marriage and therefore reduce their agency when it comes to the utilization of SRH services. Study participants postulated that female relatives, operating within the confines of patriarchy, may constrain adolescent girls’ reproductive health choices by capitalizing on this population’s generally poor understanding of menstruation and reproduction. There was a shared perception that many refugee girls enter marriage without having even a basic understanding of how vaginal intercourse is related to fertilization and pregnancy.

“There are girls, for your information, who don’t know [what sexual intercourse is] until their third or fourth night of marriage…And they won’t know until their mothers have to intervene.” (School social worker, IDI).

“The girl does not understand [how to become pregnant]. She’ll come [to the clinic] complaining that she’s been married for two months and is unable to become pregnant. But she won’t know what’s happening. She thinks one thing [about marriage], that it’s trips and white dresses and fun, and that’s it…So after two months, the problems begin, and this leads to divorce…because they still have not had sex. The girl is still a virgin. Nothing has happened between [the girl and her husband] at all.” (Obstetrician-gynecologist, Group interview).

#### Disengagement of men from care

Stakeholders perceived that men’s relative disengagement from healthcare was another deterrent to accessing contraception and SRH care among married adolescents in particular. One possible reason postulated by multiple participants is that men, who are typically the primary breadwinners of their households, have competing economic interests that prevent them from being able to accompany their wives to the clinic.

“Sometimes husbands want to come in with their wives but their only day off is a Sunday, and the clinics are closed on that day.” (Midwife, FGD).

Furthermore, healthcare providers stated that even husbands who wish to accompany their wives during clinical visits are prevented from doing so due to clinic policies. Men are deliberately excluded from physically entering clinical facilities, typically due to limited space and the wish to respect other female patients’ privacy. As such, men remain largely unexposed to family planning counseling, thereby perpetuating structures of poor health literacy, and potentially further deterring adolescent girls from seeking SRH care.

“Some husbands won’t accept IUDs because they worry it will harm them during intercourse, or could cause their wives to hemorrhage.” (Midwife, IDI).

### A conceptual framework delineating drivers of adolescent SRH needs

**Fig. 1:**
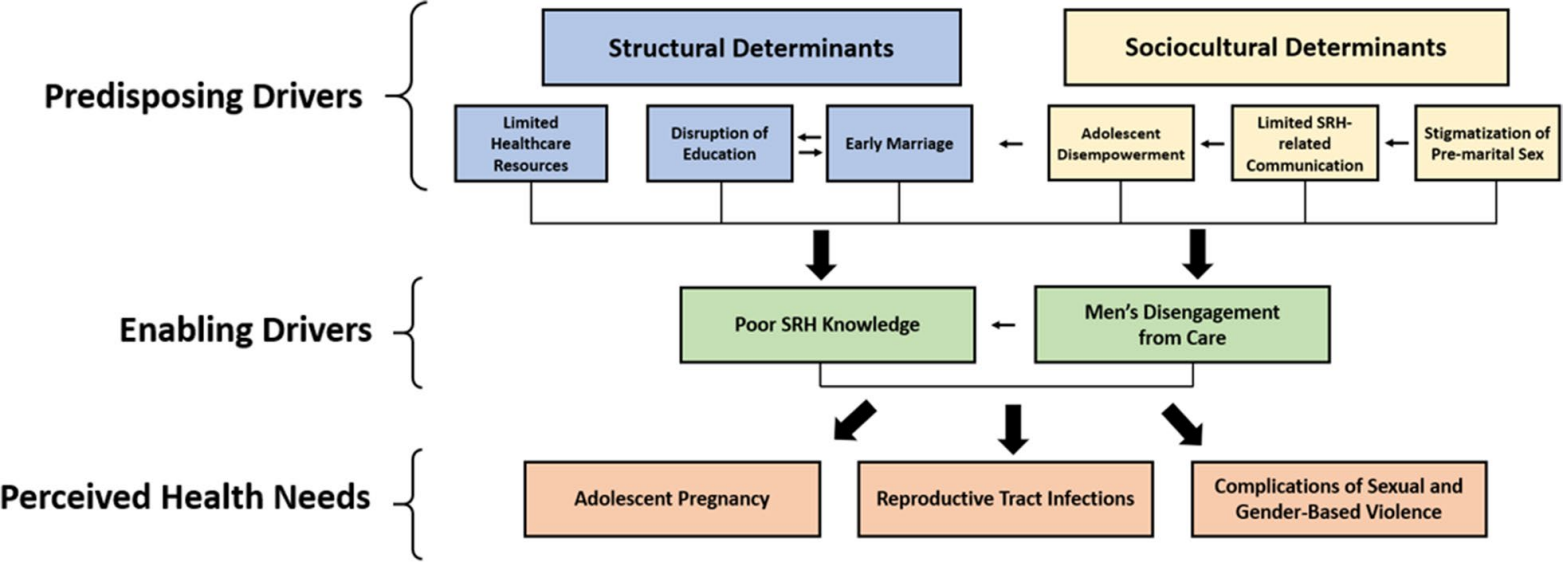
The relationships between predisposing and enabling drivers and perceived health needs

The associations between the perceived health needs and drivers identified in this study can be conceptually developed in a framework that builds upon the Gelberg–Andersen Behavioral Model to illustrate the unique interplay between gender dynamics and the sociopolitical and economic consequences of war and displacement on women’s and adolescent girls’ SRH in a conservative and patriarchal setting. Applying the three major categories described by this analytic framework to our results, we constructed Fig. 1 to illustrate the relationships and intersections of the major perceived health needs and their drivers. Though it was not possible in a small qualitative study to determine causality or draw conclusive inferences regarding the extent of influence of any one particular driver, there were clear patterns that emerged from the data that highlight areas of heightened concern and that can therefore help guide future interventions.

This framework demonstrates a range of predisposing and enabling determinants of perceived adolescent SRH needs. These are variably influenced by both displacement-related structural disparities and gender dynamics reflecting sociocultural norms that pre-date the Syrian conflict. For instance, both the lack of adequate healthcare resources and the premature termination of education are two determinants of health that can be most directly linked to displacement. Other determinants, such as stigmatization of pre-marital sex and adolescent disempowerment, are likely more reflective of conservative societal values that pre-existed conflict and forced migration, although such conditions are likely to exacerbate gender biases.

A combination of these structural and sociocultural determinants in turn influences the identified enabling drivers of health. For instance, men’s disengagement from care is likely driven both by displacement-related competing economic interests and limited healthcare resources, and also by traditional gender dynamics that preclude men’s entry into conventionally female spaces such as women’s clinics. Similarly, while school dropout has been previously demonstrated to be an independent predictor of early marriage in this context [48], our study and others suggest that additional determinants, such as adolescent and female disempowerment may also contribute to this potentially harmful practice [48, 49]. Given the qualitative nature of our study and its reliance on the perspectives of stakeholders who interface with refugee adolescent girls and women in Lebanon, it is not possible from this data to determine the extent to which displacement may have influenced many of these drivers, particularly early marriage.

Our model additionally captures the inter-dependency of the identified determinants in influencing perceived health needs. For instance, the potential incidence of both adolescent pregnancy and STIs may be associated with low rates of condom use, which in turn may be related to multiple drivers identified in this study, including insufficient healthcare resources, disengagement of men from healthcare systems, disempowerment of adolescent girls that prevents them from negotiating contraception use, and poor SRH knowledge. Indeed, stakeholders characterized lack of SRH knowledge to be pervasive and a leading contributor to poor SRH in this community. At the same time, the absence of accessible adolescent-friendly health services that are sensitive to the needs of young people has long been recognized as a barrier to adolescents’ utilization of SRH services [50]. The results of this study accordingly suggest that community, and at times educator and healthcare provider, attitudes towards adolescent SRH contribute to adolescent disempowerment and lack of care-seeking.

## Discussion

Educators and healthcare providers who provide SRH information and services to adolescent refugees are uniquely positioned to help guide the development of an early marriage intervention in this community. Building upon the drivers of poor SRH identified in this study, the relationships delineated in our proposed model, and a consultation of the literature, we consider intervention approaches that respond to the SRH needs of both married and unmarried adolescent refugee girls within a patriarchal and conservative context.

### Engage family members who make decisions about adolescent girls’ health

Study participants emphasized that an effective adolescent SRH intervention in this community would require the explicit buy-in of primary decision-makers over adolescent girls’ health: mothers and husbands. Many participants suggested prioritizing parental recruitment and sensitization prior to involving adolescents in an SRH intervention as a means of promoting community interest in and acceptance of the intervention. Alluding to the critical role that mothers play in perpetuating misinformation among adolescents, participants recommended holding separate informational sessions for mothers on basic topics related to reproductive health, as well as interactive discussions to improve and normalize communication with their daughters on sensitive topics. Such an approach is supported by evidence gathered in other contexts. Interventions addressing parent-adolescent communication on SRH-related matters have been previously shown to improve adolescent SRH outcomes including contraceptive use, sexual activity, communication with sexual partner, and perceived self-efficacy to negotiate safe sex [51, 52].

Due to competing economic interests that may limit Syrian refugee men’s interactions with the healthcare system, several respondents recommended conducting outreach activities to informally engage with men in community spaces where they typically congregate, such as coffee shops and public areas in camps. They said that discussions should specifically explore power dynamics and husbands’ beliefs regarding contraception. Motivations behind men’s restriction of contraception need further clarification in our context, as peer-reviewed studies suggest that these are likely setting- and population-dependent. Previous literature on family planning interventions in conservative settings has demonstrated that interventions that fail to address these potentially unique considerations may not be as effective in eliciting behavioral change. For instance, a quasi-experimental evaluation of an intervention to increase family planning uptake among over 3,000 Afghani women demonstrated that the primary objection to contraception use in this setting was not based in religion, as study investigators had initially hypothesized, but rather husbands’ concerns regarding the safety of contraception use and risk of causing infertility [53]. After addressing these misconceptions, study investigators demonstrated that men supported their wives’ use of contraception.

Other SRH interventions implemented in similar settings in the Middle East and North Africa have also demonstrated clear benefits to women’s health generated through the deliberate inclusion of men. For instance, a quasi-experimental evaluation of the impact of antenatal counseling on couples’ use of contraception in Egypt found that 68% of women who attended counseling sessions with their husbands were using contraception within 48 h of child delivery versus only 30% of women who attending counseling sessions alone (χ^2^ 19.50; p < 0.01) [54].

It is critical, therefore, to clarify Syrian refugee men’s attitudes on contraception use in our context, as these must be explicitly addressed by any intervention that seeks to improve adolescents’ accessibility to and utilization of contraception. Meaningfully engaging with husbands may help address several of the identified SRH determinants, including men’s disengagement from healthcare, adolescent disempowerment, and poor SRH knowledge.

### Develop a multi-pronged community-based approach to address health determinants

This study identified a number of inter-related health determinants rooted in vulnerability. Building upon the Gelberg–Andersen Behavioral Model for Vulnerable Populations, we developed a framework that links these drivers with perceived health needs and presents pathways to inform potential interventions. The complexity of the health determinants captured in our conceptual model necessitates a multi-pronged approach when developing any adolescent SRH intervention in this context.

Integrated SRH interventions that comprehensively address determinants of health and health behaviors have been shown to be effective at improving adolescent SRH in related settings. A review of early marriage interventions in low-income countries found that rigorously-designed interventions that addressed multiple drivers of early marriage, including cultural norms and empowerment, were more likely to be effective at sustainably preventing early marriage than those adopting a unidimensional approach [55]. A peer-led SRH intervention may be one effective approach in our setting, as our results suggest that adolescent Syrian refugee girls may rely upon peers for SRH information. Such an approach is supported by a recent community-based adolescent SRH intervention in India, which similarly demonstrated that a multi-pronged approach that included the delivery of a peer-educator-led rights-based SRH educational curriculum, “safe spaces” in the community for adolescents to convene, and deliberate engagement with adult community members, improved SRH outcomes by reducing early marriage and adolescent pregnancy [56].

Adolescent SRH interventions targeting Syrian refugees in Lebanon should similarly be designed to comprehensively identify and respond to community priorities. Interventions in this context should focus on adolescent empowerment by addressing several of the drivers identified in this study, including early marriage, limited educational opportunities, and patriarchal norms regulating access to health information and services.

### Recognize and address provider bias when designing adolescent-friendly services

Stigmatization of pre-marital sex emerged as an important driver of adolescent SRH in this study and manifested at times as bias exhibited by study participants themselves. Many stakeholders echoed common social understandings that certain forms of SRH education and services, including contraception, should be exclusively reserved for married, typically uniparous or multiparous adolescents. Other subtle expressions of bias could also be deduced in some respondents’ statements that engage in otherization of refugees, particularly when describing perceived values.

Provider biases in SRH provision to adolescents, for example in restricting access to certain contraceptive methods based on age, parity or marital status, have been found in numerous contexts [39, 57]. To effectively address such biases, studies have found that more is needed than simply the provision of evidence-based guidelines [57]. More recent approaches to provider bias emphasize judgement-free, social and behavioral changes that require a meaningful evaluation of the implicit beliefs underlying biases that may reflect broader sociocultural norms [57].

In settings of conflict and displacement, provider biases may reflect tensions between host and refugee communities and be driven by xenophobic stereotyping of values and behaviors. This was apparent among several participants in our study, particularly when describing their perceptions of Syrian refugees’ priorities related to gender and education. Importantly, prior studies have shown that fear and experience of such bias and mistreatment in other contexts affects SRH care-seeking behaviors [58]. Adolescent SRH interventions among Syrian refugees in Lebanon should thusly explicitly address bias in healthcare settings by working with providers to recognize the origins of such beliefs, which may be rooted in sectarianism, nationalism, and patriarchy.

### Limitations and strengths

This study has several limitations. The first is related to a fairly small sample size. Though we had initially planned to conduct at least four FGDs, we were unable to do so due to participant unavailability, which was primarily related to the small number of health facilities and providers in the region. However, we compensated by conducting a number of group interviews and were nonetheless able to attain data saturation, indicated by fewer codes generated toward the end of the coding process.

Secondly, many of the drivers contributing to poor SRH that emerged in this study are not unique to refugee populations. Despite the availability of nationally representative data in pre-conflict Syria, a paucity of comprehensive data in Lebanon challenges any conclusive determinations regarding the impact of displacement on many of the determinants identified in this study, particularly early marriage.

Additionally, though our study is specific to the health needs of adolescent refugee girls, much of the data provided was more broadly reflective of refugee women’s SRH. This speaks to the fact that, despite their vulnerability, adolescent refugees are still not recognized by many stakeholders as a distinct population at unique risk of poor health. Relatedly, because adolescent girls themselves were not included in this study, certain assumptions, particularly in relation to adolescent disempowerment, were made based on the perspectives of study participants, which are understandably influenced by their age, nationality, and profession. Our study team is conducting an ongoing qualitative study with adolescent girls in this community to address this limitation. The exclusion of adolescent boys from this study is another notable limitation; as our planned intervention specifically targets adolescent girls, addressing the needs of boys was beyond the scope of the study. Yet male Syrian refugees are an under-studied population that likely faces unique sexual and reproductive health needs that warrant dedicated attention in future research.

This study also has a number of strengths. Ours is the first to explore provider bias in the provision of SRH information and services to adolescent Syrian girls displaced in Lebanon. Another major strength of this study is that it highlights how a diversity of factors, and not just those related to displacement, may influence SRH in this population. Sociocultural, economic, and other health determinants that pre-date conflict and displacement are often overlooked in the literature on refugees and migrants, despite the fact that SRH outcomes have been shown to be strongly associated with these pre-displacement determinants, which often persist post-displacement [36]. Our model illustrates the complex relationships of both structural and sociocultural determinants contributing to perceived SRH outcomes in a patriarchal, humanitarian setting.

## Conclusions

The primary objective of this qualitative study was to explore provider and educator perspectives of the SRH services accessible to a displaced adolescent population and to determine how to effectively engage with this community to meet its health needs. This study was conducted in the context of a larger interventional study that seeks to reduce early marriage and improve access to health information and services among adolescent Syrian refugee girls in Lebanon.

Importantly, we demonstrated that stakeholders believe there to be a high burden of adolescent pregnancy, RTIs, and SGBV in this population, and that these conditions are influenced by several, inter-dependent structural and sociocultural determinants. Adolescent SRH interventions of Syrian refugees in Lebanon should engage primary decision-makers, identify and address husbands’ priorities, and respond to several of the proposed health determinants, including early marriage, adolescent and gender-based disempowerment, and poor health knowledge.

Adolescents in displacement are an especially vulnerable and often underrecognized population. Rigorously derived interventional data is urgently needed to help understand the health needs of this population and how to best meet these needs in humanitarian settings.

## Data Availability

The de-identified transcripts used in this current study are available from the corresponding author on reasonable request.

## Abbreviations

FGD: Focus group discussion
IDI: In-depth interview
IPV: Intimate partner violence
RTIs: Reproductive tract infections
SGBV: Sexual- and gender-based violence
SRH: Sexual and reproductive health
STIs: Sexually transmitted infections
